# Quantifying the effect of shade on cuticle morphology and carbon isotopes of sycamores: present and past

**DOI:** 10.1002/ajb2.1772

**Published:** 2021-12-31

**Authors:** Joseph N. Milligan, Andrew G. Flynn, Jennifer D. Wagner, Lenny L.R. Kouwenberg, Richard S. Barclay, Bruce W. Byars, Regan E. Dunn, Joseph D. White, Bernd Zechmann, Daniel J. Peppe

**Affiliations:** ^1^ Terrestrial Paleoclimatology Research Group, Department of Geosciences Baylor University Waco TX USA; ^2^ Department of Integrative Biology University of California Berkeley, and UC Museum of Paleontology Berkeley CA USA; ^3^ Department of Geology Field Museum of Natural History Chicago IL USA; ^4^ Department of Paleobiology National Museum of Natural History, Smithsonian Institution, 10th & Constitution Avenue NW Washington D.C. USA; ^5^ Center for Spatial Research, Baylor University Waco TX USA; ^6^ Natural History Museums of Los Angeles County, La Brea Tar Pits Los Angeles CA USA; ^7^ Department of Biology Baylor University Waco TX USA; ^8^ Center for Microscopy and Imaging Baylor University Waco TX USA

**Keywords:** daily light integral, fossil, leaf carbon isotope, leaf cell area, paleobotany, Platanaceae, *Platanus*, undulation index

## Abstract

**Premise:**

Reconstructing the light environment and architecture of the plant canopy from the fossil record requires the use of proxies, such as those derived from cell wall undulation, cell size, and carbon isotopes. All approaches assume that plant taxa will respond predictably to changes in light environments. However, most species‐level studies looking at cell wall undulation only consider “sun” or “shade” leaves; therefore, we need a fully quantitative taxon‐specific method.

**Methods:**

We quantified the response of cell wall undulation, cell size, and carbon isotopes of *Platanus occidentalis* using two experimental setups: (1) two growth chambers at low and high light and (2) a series of outdoor growth experiments using green and black shade cloth at different densities. We then developed and applied a proxy for daily light integral (DLI) to fossil *Platanites* leaves from two early Paleocene floras from the San Juan Basin in New Mexico.

**Results:**

All traits responded to light environment. Cell wall undulation was the most useful trait for reconstructing DLI in the geological record. Median reconstructed DLI from early Paleocene leaves was ~44 mol m^−2^ d^−1^, with values from 28 to 54 mol m^−2^ d^−1^.

**Conclusions:**

Cell wall undulation of *P. occidentalis* is a robust, quantifiable measurement of light environment that can be used to reconstruct the paleo‐light environment from fossil leaves. The distribution of high DLI values from fossil leaves may provide information on canopy architecture; indicating that either (1) most of the canopy mass is within the upper portion of the crown or (2) leaves exposed to more sunlight are preferentially preserved.

Many plant traits are sensitive to light intensity, with changes in light affecting almost all parts of plant development, including anatomy and morphology, chemical composition, physiology, and growth and reproduction (Poorter et al., 2019). Further, light environments also strongly influence terrestrial ecosystem structure, composition, and climate (Betts et al., 1997; Asner et al., 2003). Paleobotanists have used light‐sensitive plant traits to differentiate leaves from different light environments in the fossil record (i.e., sun and shade leaves; e.g., Kürschner, 1997; Guignard et al., 2001; Wu et al., 2009; Maslova and Shilin, 2011; Xiao et al., 2011; Maslova et al., 2018; Wang et al., 2018) and to reconstruct canopy structure of ancient forests (Crifò et al., 2014; Dunn et al., 2015b; Bush et al., 2017; Graham et al., 2019). There are two major reasons for reconstructing the light environment in the past. First, variation in leaf traits due to light environment can influence atmospheric, ecological, and environmental proxies that use fossil leaves (e.g., Reichgelt and D'Andrea, 2019). Therefore, any proxy that is in part dependent on modern calibrations (e.g., Royer et al., 2007; Peppe et al., 2011; Schubert and Jahren, 2012; Utescher et al., 2014; McElwain and Steinthorsdottir, 2017; Soh et al., 2017; Konrad et al., 2020) may be impacted by the light environment. Second, canopy structure is a critical component of ecosystems. Changes in canopy structure can influence the Earth's climate (Bastable et al., 1993; Kala et al., 2014) and play an important role in ecological interactions (Kay et al., 1997; Asner et al., 2003; Dunn et al., 2015b). Therefore, canopy structure is used in many ecological and climate modeling studies (Running and Gower, 1991; Betts et al., 1997; Kala et al., 2014; Mahowald et al., 2016; White et al., 2020).

Specifically, the traits used by paleobotanists to reconstruct light environment include the size and/or morphology of leaf pavement epidermal cells (Kürschner, 1997; Wu et al., 2009; Maslova and Shilin, 2011; Xiao et al., 2011; Dunn et al., 2015b; Bush et al., 2017; Maslova et al., 2018; Wang et al., 2018), leaf vein density (Crifò et al., 2014; Londoño et al., 2018), leaf mass per area (Turney et al., 2002), leaf carbon isotopes (Bush et al., 2017; Graham et al., 2019), stomatal density (Kürschner, 1997), leaf morphology (Nguyen Tu et al., 2004; Maslova et al., 2015), cuticle ultrastructure (Guignard et al., 2001), and in situ stump density and size (Williams et al., 2003; Michel et al., 2014). Recent efforts have also attempted to incorporate multiple traits in both modern (Cheesman et al., 2020) and fossil plants (Bush et al., 2017). Here we focused exclusively on three of these traits that can be measured simultaneously on fossil leaves: the size and morphology (undulation) of leaf pavement epidermal cells and leaf carbon isotopes.

The waviness of leaf epidermal cell junctions, typically referred to as undulations, form due to differential cytoskeletal patterning (Fu et al., 2005) and have been hypothesized to provide a myriad of functions including more efficient chemical signaling, increased epidermal integrity, increased epidermal flexibility, and reduced mechanical stress (reviewed by Vőfély et al., 2018 and sources cited within). Leaf epidermal cells grown in low‐light environments are larger (Watson, 1942; Carins Murphy et al., 2016) and have more‐undulated (wavy) anticlinal cell walls than on leaves grown in higher light (Watson, 1942). These differences in leaf epidermal cells are thought to be a consequence of the rate of cuticle hardening, with higher irradiance causing more rapid cuticle hardening and thus less time for epidermal cell expansion and differential expansion of cell walls (Watson, 1942). The degree of anticlinal epidermal cell wall waviness was first formally quantified and named by Kürschner (1997) as undulation index (UI). Several studies have used UI to separate sun and shade morphotypes for a variety of plant taxa from the fossil record (Kürschner, 1997; Wu et al., 2009; Xiao et al., 2011; Bush et al., 2017; Wang et al., 2018). Dunn et al. (2015b) built off these observations to develop a community‐based, taxon‐free approach for reconstructing canopy architecture by using the size and UI of epidermal phytoliths (silica bodies derived from epidermal cells). The study by Dunn et al. improved upon traditional methods by fully quantifying the light habitat, using leaf area index (LAI), which is a measure of canopy structure, and demonstrated that at higher LAI (i.e., in light environments with more shade), phytoliths were larger and more undulated.

Despite its utility, UI has some important deficiencies in the understanding and implementation of its use as a proxy for light environment. First, while cell size and morphology likely operate on a spectrum of light values (see Poorter et al., 2019), most studies do not quantify the terms “sun” and “shade” (e.g., Kürschner, 1997), limiting this proxy to a qualitative approach of light environment. Second, studies are inconsistent on whether the abaxial (lower) or adaxial (upper) surface is used to calculate UI (see review by Bush et al., 2017). Because cell morphology differs between the two surfaces (Vőfély et al., 2019), it is not clear whether the light–UI relationship is the same on both sides of the leaf. Third, the relationship between cell morphology and light appears to be species‐specific (Cheesman et al., 2020), suggesting that for an individual species or morphotype from the fossil record, modern calibration may be required. Fourth, light experiments on *Arabidopsis thaliana* (Thomas et al., 2004) and five species of grasses (Dunn et al., 2015a) have confirmed that light quantity plays an important role in determining cell morphology. To date, however, no study has addressed the role of light quality on epidermal cell morphology. Of particular importance is the red to far red (R/FR) ratio, which varies throughout the canopy (Smith, 1982) and can influence plant development (e.g., Griffith and Sultan, 2005).

Leaves that experience lower irradiance are depleted in δ^13^C compared to leaves that experience higher irradiance. These differences in leaf δ^13^C have been widely observed in light experiments (Lynch et al., 2012), within the crown of a single tree (Le Roux et al., 2001; Xiao et al., 2013), and throughout the forest canopy (Broadmeadow et al., 1992; Turney et al., 2002; Graham et al., 2014; Cheesman et al., 2020). Leaf δ^13^C differs because at low light, photosynthesis is reduced and the ratio of internal to external CO_2_ concentration (ci/ca) is elevated, resulting in increased isotopic discrimination of δ^13^C (Farquhar et al., 1989). Additionally, within a closed forest canopy, vertical gradients in light, humidity, atmospheric CO_2_ concentration, and δ ^13^C of the atmosphere (δ^13^C_atm_) can all contribute to differences in δ ^13^C at the leaf level (δ^13^C_leaf_), known as the canopy effect (e.g., Graham et al., 2014). Differences in δ^13^C_leaf_ between fossil sun and shade morphotypes have been observed in some studies (Turney et al., 2002; Nguyen Tu et al., 2004; Xiao et al., 2013) although differences are not always statistically significant (e.g., Xiao et al., 2013). Graham et al. (2014) developed and applied (Graham et al., 2019) a community‐based approach by using the range of δ^13^C_leaf_ from leaf litter to ascertain whether a canopy was open or closed.

Due to limitations and uncertainties inherent in proxies, it is often ideal to compare or incorporate multiple proxies. However, where multiple traits such as cell area, UI, δ^13^C_leaf_, have been examined in detail, the results are mixed. For example, Bush et al. (2017) found no correlation between UI and δ^13^C_leaf_ from legumes of the early Miocene Mush Valley site in central Ethiopia. They suggested that possible factors other than light (e.g., temperature, relative humidity, wind speed) could be controlling one or both traits. Cheesman et al. (2020) evaluated the response of cell area, UI, and δ^13^C_leaf_ to LAI for four species from the Daintree Rainforest, Australia. While all species responded to an increase in LAI with an increase in cell area and more negative δ^13^C_leaf_, only a few species increased their UI; with two species showing no UI response. These results highlight the need for more work detailing and understanding the UI response on modern species to differences in light environments.

Here we quantified the response of cell size and morphology and carbon isotopes of *Platanus occidentalis* L. (Platanaceae) using two different experimental setups. We focused on *Platanus* in this study because (1) it commonly grows in deciduous broadleaf forests, and these types of forests exhibit a range of LAI values (Dunn et al., 2015b); and (2) epidermal cells of *Platanus acerifolia* (Aiton) Willd. respond to differences in crown position (Maslova et al., 2008), although UI has not been quantified; and (3) Platanaceae are ubiquitous in the Cenozoic (e.g., Kvaček et al., 2001; Pigg and DeVore, 2010) and early Cretaceous record across Laurasia (e.g., Upchurch, 1984; Crane et al., 1993; Wang et al. 2011). In this study, we used two experimental setups. The first involved two growth chambers, one at low and one at high light to assess light quantity. The second setup consisted of an outdoor field experiment of green and black shade cloth of different densities to assess both light quantity and quality. We measured δ^13^C_leaf_ to understand variations of carbon isotopes across light environments and cell morphology traits on both the abaxial and adaxial surface of the leaf to quantify changes in cell size and UI. Based on these examined traits, we then developed a proxy for daily light integral (DLI), the number of photosynthetically active photons (400–700 nm), integrated over a day, and applied the proxy to two early Paleocene floras from the San Juan Basin in New Mexico, United States.

## MATERIALS AND METHODS

### Growth chamber experiment

**Figure 1:**
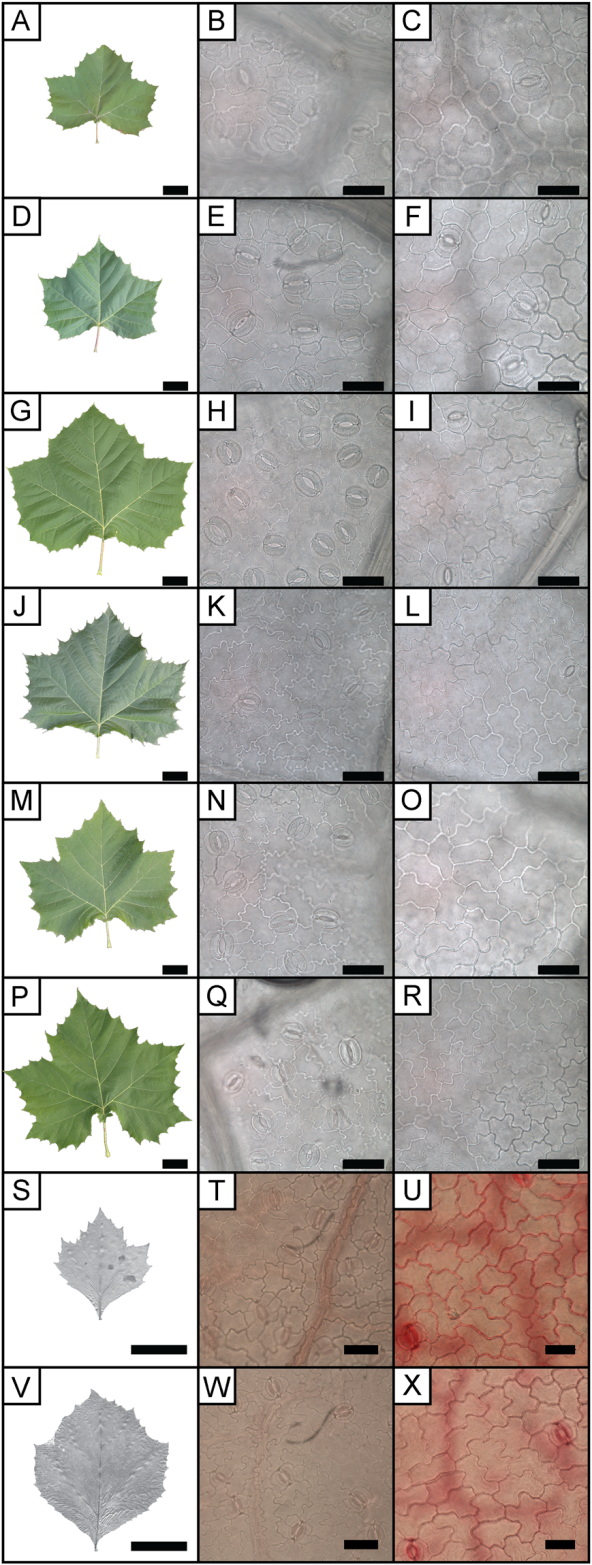
Example images of leaf and epidermal cells from *Platanus occidentalis*. Leaf (A, D, G, J, M, P, S, V), abaxial (B, E, H, K, N, Q, T, W), and adaxial (C, F, I, L, O, R U, X) epidermis of leaves from the shade cloth experiment (A–R) and growth chamber experiment (S–X). No shade cloth (A–C), 30% black shade cloth (D–F), 60% black shade cloth (G–I), 90% black shade cloth (J–L), 60% green shade cloth (M–O), 87% green shade cloth (P–R), high‐light growth chamber (S–U), low‐light growth chamber (V–X). Leaf scale bars are 5 cm; epidermal scale bars are 50 µm. For epidermal images, the mesophyll tissue was cleared using NaOH or KOH. Additionally, leaves from the growth chamber experiment were stained with safranin O. (T, U, W, X)

We conducted an enclosed growth chamber experiment to assess the effect of changes in light quantity on *P. occidentalis* at The Field Museum of Natural History, Chicago, Illinois, United States. In this experiment, we used leaf material from the same light experiment, with *P. occidentalis* saplings, as the study of Lynch et al. (2012). Saplings were potted with a mixture of peat moss, perlite, and sand (2:1:1) and grown from April to July 2008 in two Conviron E8 growth chambers (Conviron, Winnipeg, Canada). Five saplings were grown in each chamber. Plants were watered twice a week with an automated sprinkler system. Growth light levels (photosynthetically active radiation; PAR) were either 230 ± 82 (1σ) or 20.4 ± 18 (1σ) µmol m^−2^ s^−1^ depending on the chamber and the height of the plant. All other environmental variables were held constant between the two chambers. During the experiment, the average daily temperature was 16.6 ± 3.3°C, and the average daily photoperiod was 14 h with a 30‐min simulated dawn and dusk. CO_2_ was set to ~500 ppm, and relative humidity varied between 70% and 85%. The δ^13^C_atm_ was not measured during this experiment. Five leaves from each sapling (Figure 1) were used for isotope and cuticle morphology analysis (*N* = 25 per light treatment).

### Field shade cloth experiments

To assess the effects of light quantity and quality, ~1.0–2.0‐m tall bareroot saplings of *P. occidentalis* were planted in January 2018 at the Lake Waco Wetlands, Waco, Texas, United States (Appendix S1). The saplings were grown from seeds at Texas Pecan Nursery Inc. (Chandler, TX, USA) and were not clones. The leaves used in this study were from leaf growth that developed from March to August 2018. The field experiment consisted of six, 3.0 × 3.0 m plots, with five saplings within each plot. Five of the plots contained a 3.0 × 3.0 × 3.0 m PVC structure covered by different types of shade cloth, with a sixth as control. Shade cloth was 30%, 60%, 90% black neutral‐density cloth and 60% and 87% green cloth (Hummert International, Earth City, MO, USA). Different color shade cloth was used to assess variability in light quality, with green shade cloth used to mimic low R/FR ratios as seen in natural canopies (e.g., Griffith and Sultan, 2005). In the control plot, five samplings were grown within a 3.0 × 3.0 m plot adjacent to the shade‐cloth PVC structures. Plants were watered twice weekly.

Temperature, relative humidity, and solar radiation were measured throughout the duration of the experiment by a local weather station (DW7589) in 5‐min intervals. Values from the weather station were corrected for each light treatment based on the shade cloth properties. For solar radiation, values were corrected based on the density of the shade cloth (percentage). For example, because the 60% shade cloth only allows 40% of light to pass through, solar radiation from DWS7589 was multiplied by 0.4. For temperature and relative humidity, values were corrected based on the relative difference between the weather station data and light treatment, based on measurements made by HOBO data loggers (Onset Computer Corp, Bourne, MA, USA) placed within the shade structures for a subset of the experiment (May–June). Average daily temperature and relative humidity varied depending on light treatment; however, this variation was less than 6% (Table 1).

**Table 1:** Average daily environmental attributes during each experiment and number of leaves analyzed (N). Five plants were used for each treatment

Setup	Treatment (µmol m^−2^ s^−1^/% shade cloth)	Daily light integral (mol m^−2^ d^−1^)	Temperature (°C)	Relative humidity (%)	*N*
Growth chamber	High (230)	11.7 ± 1.0	16.6 ± 3.3	70−85	25
	Low (20.4)	4.1 ± 0.9	16.6 ± 3.3	70−85	25
Shade cloth	Control (0%)	45.5 ± 13.7	25.7 ± 7.3	56.5 ± 18.0	10
	Black (30%)	31.9 ± 9.6	24.9 ± 7.2	57.2 ± 17.6	10
	Black (60%)	18.2 ± 5.5	24.3 ± 7.3	56.2 ± 18.0	10
	Green (60%)	18.2 ± 5.5	24.2 ± 7.2	60.8 ± 17.5	9
	Green (87%)	5.9 ± 1.8	24.1 ± 7.2	56.7 ± 17.9	9
	Black (90%)	4.6 ± 1.4	24.3 ± 7.2	54.5 ± 17.5	10

One to two leaves from each sapling per light treatment were used for isotope and cuticle morphology analysis (*N* = 9–10 per light treatment; Figure 1). All leaves were sampled from the top of the tree crown to ensure only new growth was sampled and to avoid bias from self‐shading.

### Fossil locality

**Figure 2:**
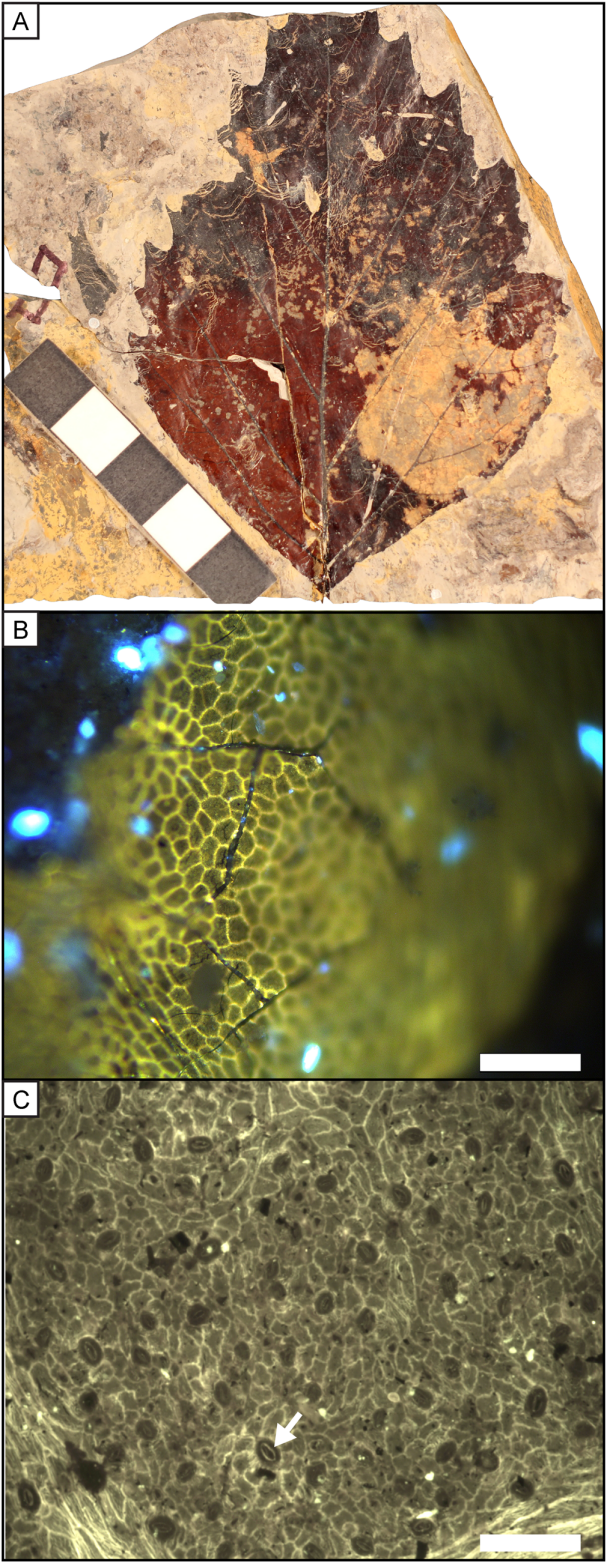
Example images of a *Platanites* sp. leaf (A) and adaxial (B) and abaxial (C) epidermal cells. Leaf scale bar is 5 cm and epidermal scale bars are 100 µm. White arrow (C) points to cuticular ledge on stoma

Fossil *Platanites* leaves (Figure 2A) were collected from two sites in the San Juan Basin, New Mexico: the De‐Na‐Zin Wilderness Area (site DP1304) and Kimbeto Wash (site DP1311). The age of the sites was calculated to be 65.33 ± 0.05 Ma (DP1304) and 64.60 ± 0.01 Ma (DP1311) using sediment accumulation rates based on the local stratigraphic position of magnetostratigraphic boundaries (Flynn, 2020; Flynn et al., 2020). Fossils from site DP1304 were collected from interbedded muds and sands, which we interpret to have been stacked crevasse splay deposits. DP1311 was collected from a carbonaceous shale interpreted to have been a ponded crevasse splay or overbank deposit. Mean annual temperature (MAT) and mean annual precipitation (MAP) reconstructions for site DP1304 and DP1311 are based on binned leaf collections from 65.40–64.96 Ma and 64.67–64.40 Ma, respectively. For site DP1304, MAT is estimated at 20.4 ± 4.0°C with an estimated MAP of 183.0 + 150.4/–82.6 cm/yr (Flynn, 2020). For site DP1311 MAT is estimated at 19.8 ± 4.0°C with an estimated MAP of 188.9 + 155.3/–85.2 cm/yr (Flynn, 2020). These MAT and MAP estimates are indistinguishable from each other, indicating the two sites sample similar climates. Fossils were assigned to the genus *Platanites* based on foliar architecture and leaf venation (e.g., Flynn and Peppe, 2019; Flynn, 2020). In addition, stomata have a raised ledge over the guard cell, which is characteristic of modern Platanaceae (Carpenter et al., 2005) (Figure 2C). For cuticle morphology analysis, 52 leaves from site DP1304 and 20 leaves from site DP1311 were used, and 37 of those same leaves from site DP1304 and 15 leaves from site DP1311 were also used for isotope analyses.

### Cell size and morphology

On extant leaves, an approximately 1‐cm^2^ section was cut between the midvein and margin. Leaves were macerated using common chemicals for removing mesophyll tissue: 5% KOH (growth chamber experiment) and 5% NaOH (shade cloth experiment). Maceration took 1–7 days and KOH/NaOH was replaced when discolored. Samples were then placed in household bleach for 1–5 min to clear the tissue. Leaves from the growth chamber experiment went through a dehydration series (25%, 50%, 100% ethanol) before being stained with safranin O. All samples were water mounted for light microscopy, and three field of views on both the adaxial and abaxial side were imaged at 400× magnification with either a Leica DM2500 (Leica Microsystem, Buffalo Grove, IL, USA) and Nikon DS‐Fi1 camera (growth chamber experiment) or Olympus BX51 microscope (Olympus, Center Valley, PA, USA) and Leica DFC450 digital camera (shade cloth experiment) (Figure 1). Fossil leaves needed no preparation, and the adaxial surface was imaged using the Olympus BX51 microscope and Leica DFC450 digital camera with epifluorescence (420–490 nm filter cube) at 200× magnification (Figure 2B). The abaxial surface was not photographed because on most leaves it was too poorly preserved to determine epidermal cell boundaries.

We traced 10 cells per field of view (30 cells total per leaf) and measured the area and perimeter of each cell using ImageJ (http://imagej.nih.gov/ij/). Cells overlying veins, cells with shape or size that were influenced by trichomes, and subsidiary cells were avoided. Undulation index (UI) was calculated as the ratio of the cell circumference to the circumference of a circle with the same area (according to Kürschner, 1997; Eq. 1):

(1) $$\text{UI}=\frac{C_{e}}{C_{o}}=\frac{C_{e}}{2\pi \sqrt{\frac{A_{e}}{\pi }}},$$

where *C*_*e*_ (μm) is the cell circumference, *C*_0_ (μm) is the circumference of a circle with the same area as the cell, and *A*_e_ (μm^2^) is the cell area. UI for a cell that is a perfect circle is one and increases as the cell becomes wavier, independent of cell size.

### δ^13^C analysis

All specimens were analyzed for δ^13^C using the Delta‐V Advantage mass spectrometer (Thermo Fisher Scientific, Waltham, MA, USA). For δ^13^C_leaf_ analysis all extant specimens (*N* = 108) were whole punched near the same location where cuticle morphology measurements were taken. For fossil specimens, cuticle from a subset of fossil leaves (DP1304, *N* = 37; DP1311, *N* = 15) was gently scraped from the rock taking care to remove as little matrix with the cuticle as possible. The scraped material was submerged in HCl (36.5–38%) to remove carbonate, rinsed in distilled water, treated with HF (48%) to dissolve silicates, rinsed again in distilled water, and then oven dried at 60°C.

To account for differences in δ^13^C_leaf_ due to changes in δ^13^C_atm_ over time, we calculated the offset between δ^13^C_leaf_ and δ^13^C_atm_ (Δleaf; Eq. 2; Farquhar et al., 1989) as

(2) $$\Delta \text{leaf}(‰)=\frac{\delta^{13}C_{\text{atm}}-\delta^{13}C_{\text{leaf}}}{1+\delta^{13}C_{\text{leaf}}/1000}$$

For the shade cloth experiment, δ^13^C_atm_ was –8.59‰ based on the average from March to August 2018 from Mauna Loa, Hawaii (Keeling et al., 2001). In general, there is good agreement of the average δ^13^C_atm_ from March to August in 2001 and 2010 between Moody, Tx (~44 km away from the experiment) and Mauna Loa, Hawaii (Appendix S2). For the growth chamber experiment, Δleaf was not calculated because δ^13^C_atm_ was not measured. For the earliest Paleocene atmosphere, we used data from Tipple et al. (2010) to estimate δ^13^C_atm_ value of –4.97‰ for site DP1304 and –4.86‰ for site DP1311.

### Data analyses

Using leaf means, we used a one‐sample Kolmogorov–Smirnov test that showed values for all input variables are normally distributed. We then conducted a one‐way ANOVA and post hoc Tukey's test or Student's *t*‐test on leaf means to evaluate the significance of variation in UI, cell area, and δ^13^C_leaf_ among treatment groups and fossil localities. We used linear regressions to look at how traits vary by light, considering both experiments. Because the light experiments have different light setups (e.g., photoperiod, maximum PAR values), we converted the light values to DLI (mol m^−2^ d^−1^; Table 1). We note that the range in DLI found in this study, 4.1–45.5 mol m^−2^ d^−1^, is comparable to the full range of DLI measured in nature (Faust and Logan, 2018; Poorter et al., 2019). We also used linear regressions to evaluate potential relationships between adaxial and abaxial surfaces and δ^13^C_leaf_ and UI. All statistical analyses were conducted using the R base package (v.2.14.2; R Core Team, 2020).

### Shade proxy development

To reconstruct DLI from our fossils, we assessed predictive multiple linear regression models for DLI based on the individual leaf cuticle size and undulation and Δleaf from the shade cloth experiment. Model selection was made using adjusted *r* ^2^, which adjusts the *r* ^2^ value based on the number of terms used in the model, and Akaike information criterion (AIC) to mathematically evaluate how well the model fit the data. The growth chamber experiments were excluded from our predictive multiple linear regression models because Δleaf could not be determined. The DLI was predicted for each individual fossil leaf. The median and range of DLI for each locality was used to determine canopy architecture.

## RESULTS

### Cell size, morphology, and carbon isotope response to light environment

**Figure 3:**
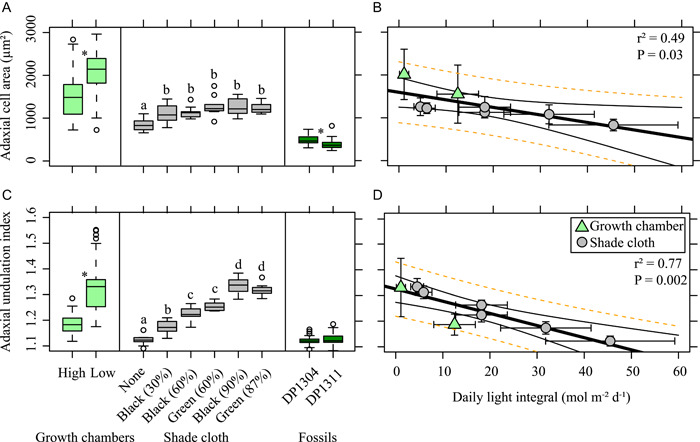
Adaxial (upper surface) cell size and morphology versus light treatment. Boxplots (A, C) are on the individual leaf level. For multiple comparisons, different letters indicate significantly different values at the 0.05 level. *P ≤ 0.05. For linear regressions of daily light integral (B, D), standard errors of the treatment means are plotted. Black lines are the 95% confidence interval; dotted orange lines are the prediction interval around the linear regression

We found a significant difference in adaxial cell area based on light treatment in both experiments (Figure 3A; growth chamber, *t* = –2.78, df = 47, *P* = 0.007; shade cloth, *F*_5,52_ = 8.22, *P* < 0.001). However, in the shade cloth experiment, the control was the only light treatment that was significantly different from the other light treatments (Figure 3A; Appendix S3). Overall, there was a significant negative correlation between DLI and adaxial cell area (Figure 1B; cell area = –17.64 × DLI + 1595.62; *r* ^2^ = 0.49, *P* = 0.03). Likewise, we found a significant difference in adaxial UI based on light treatment in both experiments (Figure 3C; growth chamber, *t* = –6.14, df = 30, *P* < 0.001; shade cloth, *F*_5,52_ = 106.2, *P* < 0.001). In the shade cloth experiment, average adaxial UI was significantly different for every light quantity (Appendix S3). Overall, there was a significant negative correlation between DLI and adaxial UI (Figure 3D; UI = –0.005 × DLI + 1.32; *r* ^2^ = 0.77, *P* = 0.002). There was no difference in adaxial cell area or adaxial UI based on light quality (Figure 3A, C).

**Figure 4:**
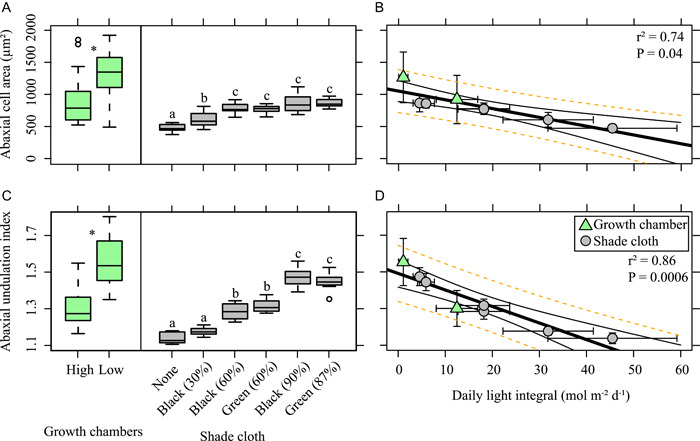
Abaxial (lower surface) cell size and morphology versus light treatment. Boxplots (A, C) are on the individual leaf level. For multiple comparisons different letters indicate significantly different values at the 0.05 level. *P ≤ 0.05. For linear regressions of daily light integral (B, D), standard deviations of the treatment means are plotted. Black lines are the 95% confidence interval; dotted orange lines are the prediction interval around the linear regression

Like the adaxial surface, we found a significant difference in abaxial cell area based on light treatment in both experiments (Figure 4A; growth chamber, *t* = –3.45, df = 48, P = 0.001; shade cloth, *F*_5,52_ = 26.63, *P* < 0.001). For the shade cloth experiment, the abaxial cell area from the no shade cloth and 30% black shade cloth treatments were significantly different from each other and all other light treatments (Figure 4A; Appendix S3). There is a significant negative correlation between DLI and abaxial cell area (Figure 4B; cell area = –13.67 × DLI + 1052.46; *r* ^2^ = 0.74, *P* = 0.04). Likewise, we found a significant difference in abaxial UI based on light treatment in both experiments (Figure 4C; growth chamber, *t* = –8.14, df = 45, *P* < 0.001; shade cloth, *F*_5,52_ = 122.7, *P* < 0.001). In the shade cloth experiment, abaxial UI was significantly different for every light quantity (Figure 4C; Appendix S3). Overall, there was a significant negative correlation between DLI and abaxial UI (Figure 2D; UI = –0.009 × DLI + 1.49; *r* ^2^ = 0.86, *P* < 0.001). There was no difference in abaxial cell area or abaxial UI based on light quality (Figure 4A, C).

The UI values from our two experiments, ranging from 1.09 to 1.80 (depending on the surface, adaxial or abaxial, and light treatment), are consistent with UI values measured on a limited number of modern plants (*N* = 12; for review, see Bush et al., 2017; Cheesman et al., 2020) which range from 1.11 in *Liquidambar formosana* (Xiao et al., 2011) to 2.28 in shade leaves of *Impatiens parviflora* (Hughes, 1959; measured by Bush et al., 2017).

**Figure 5:**
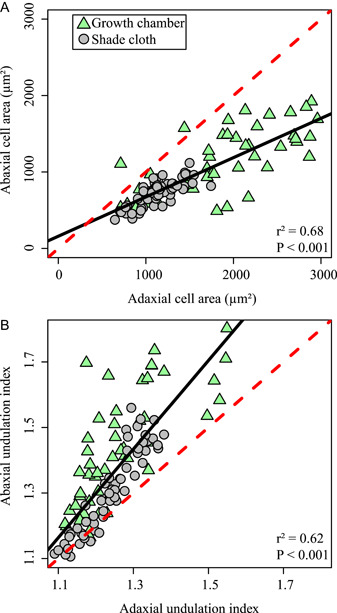
Relationship between cell morphology on the adaxial (upper) and abaxial (lower) surface. Solid black line is the linear regression through all data points. Dotted red line is the 1:1 relationship

In general, we found that the adaxial surface had larger cells that were less undulated compared to the abaxial surface. There were strong linear relationships between adaxial and abaxial cell area (Figure 5A; abaxial cell area = 0.51 × adaxial cell area + 159.97; *r* ^2^ = 0.68, *P* < 0.001) and adaxial and abaxial UI (Figure 5B; abaxial UI = 1.35 × adaxial UI – 0.32; *r* ^2^ = 0.62, *P* < 0.001). Unlike Xiao et al. (2011), we found the abaxial surface to have a greater difference in UI values between light treatments. However, while the abaxial surface was generally more undulated, there was a consistent relationship between UI between the adaxial and abaxial surface. The similarity in response to DLI between both surfaces suggest that, at least for *Platanus occidentalis*, either surface can be used for reconstructing the light environment.

**Figure 6:**
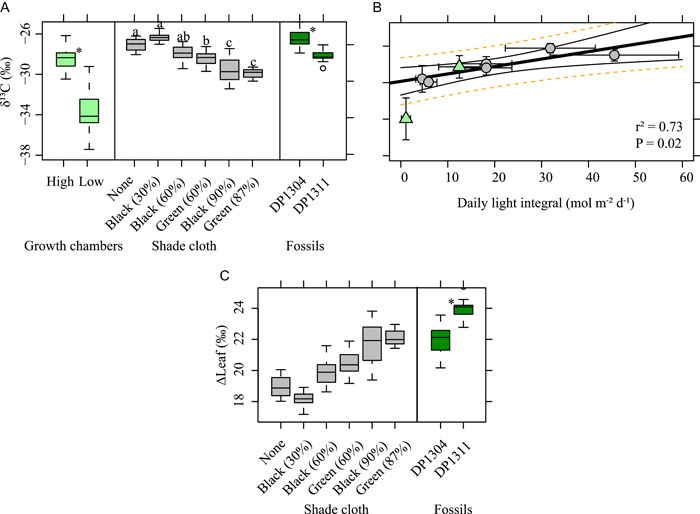
Relationship between light treatment and leaf δ^13^C (δ^13^C_leaf_). Boxplots (A) are on the individual leaf level. For multiple comparisons, different letters indicate significantly different values at the 0.05 level. *P ≤ 0.01. The linear regression of daily light integral and δ^13^C_leaf_ (B) only includes the shade cloth experiment. δ^13^C of the atmosphere (δ^13^C_atm_) was not measured in the growth chamber experiment; however, the points are included for reference (triangles). In the linear regression, standard deviations of the site means are plotted. Black lines are the 95% confidence interval and the dotted orange lines are the prediction interval around the linear regression. (C) Linear regression of daily light integral and Δleaf, the carbon isotope discrimination in plant leaves that considers δ^13^C_atm_; see Eq. 2 in the text

In both experiments, lower light treatments resulted in more negative δ^13^C_leaf_ values compared to higher light treatments (Figure 6A). In the shade cloth experiment, there was no difference in δ^13^C_leaf_ based on light quality (Figure 6A; Appendix S3). There was a significant negative correlation between δ^13^C_leaf_ and DLI for the shade cloth experiment (Figure 6B; δ^13^C_leaf_ = 0.08 × DLI – 29.86; *r* ^2^ = 0.73, *P* = 0.02). In addition, Δleaf followed the same pattern as δ^13^C_leaf_ and ranged from an average of 18.13‰ in the 30% black shade cloth treatment to an average of 21.67‰ in the 90% black shade cloth treatment.

**Figure 7:**
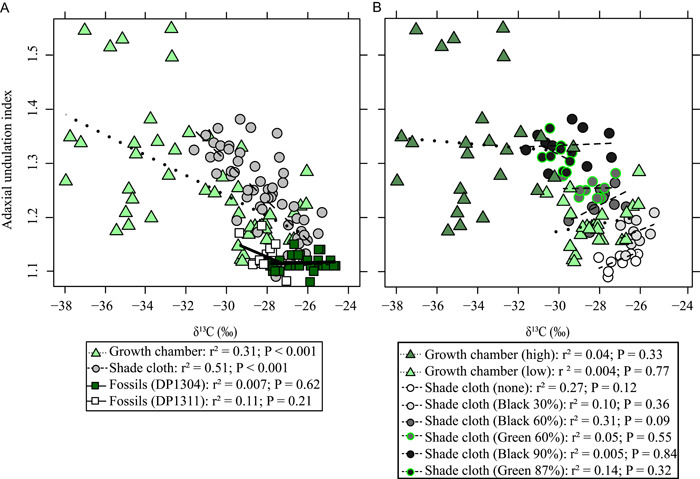
Relationship between leaf δ^13^C and adaxial undulation index. (A) Linear regression based on experiment or fossil data set. (B) Linear regressions based on light treatment within each experiment

Adaxial UI was correlated with δ^13^C_leaf_ on modern specimens (Figure 7A), but the strength of the relationship was dependent on the experiment (shade cloth, *r* ^2^ = 0.51, *P* < 0.001; growth chamber, *r* ^2^ = 0.31, *P* < 0.001). However, we found no correlation between adaxial UI and δ^13^C_leaf_ at the individual treatment level (Figure 7B; *P* > 0.05). If these relationships hold true for our fossil leaves, then the lack of adaxial UI and δ^13^C_leaf_ correlation indicates that these leaves are all from a similar light environment (DP1304, *r* ^2^ = 0.007, *P* = 0.62; DP1311, *r* ^2^ = 0.11, *P* = 0.21).

### Predictive DLI linear regression

Based on the multiple linear regression models from the shade cloth experiment (Table 2), the best models (lowest AIC, highest *r* ^2^, and lowest SE) for reconstructing DLI included UI, cell area, and Δleaf (model 1, *r* ^2^ = 0.91, SE = 4.45 mol m^−2^ d^−1^) or second order polynomial of UI (model 2; ΔAIC = 2.24, *r* ^2^ = 0.90, SE = 4.56 mol m^−2^ d^−1^). The model that included only cell area (model 8; ΔAIC = 110.03, *r* ^2^ = 0.36, SE = 32.47 mol m^−2^ d^−1^) was the worst model. These results are not unsurprising due to the lack of significant differences between light treatments from 30% to 90% black shade cloth (Figure 3A).

**Table 2:** Regression models for predicting daily light integral (DLI). Data is based on 58 leaves from the shade cloth experiment only. Leaves from the growth chamber experiment were excluded because δ^13^C of the atmosphere was not measured; therefore, Δleaf cannot be directly compared between the two. Note that all cuticle morphology is based on the adaxial surface only. SE is the standard error in mol m^−2^ d^−1^. Abbreviations: undulation index (UI), cell area (CA), difference of the AIC score of each model from the best model (ΔAIC), carbon isotope discrimination in plant leaves (Δleaf)

Model	Equation	*r* ^2^	SE	*F*	*P*	ΔAIC
1	DLI = –136.60 × UI – 0.0010 × CA – 1.38 × Δleaf + 228.90	0.91	4.45	185.1	<0.001	—
2	DLI = –1104.10 × UI + 377.40 × UI^2^ + 806.80	0.90	4.56	260.9	<0.001	2.24
3	DLI = –157.30 × UI – 0.010× CA – 226.80	0.90	4.69	246.9	<0.001	5.14
4	DLI = –151.99 × UI – 1.38 × Δleaf + 236.64	0.89	4.87	227.7	<0.001	9.35
5	DLI = –172.78 × UI + 234.50	0.88	5.08	412.8	<0.001	13.32
6	DLI = –0.023 × CA – 5.40 × Δleaf + 156.28	0.68	8.29	60.49	<0.001	71.10
7	DLI = –6.65 × Δleaf + 154.97	0.56	9.68	7315	<0.001	88.09
8	DLI = –0.038 × CA + 63.73	0.36	11.69	32.47	<0.001	110.03

### Reconstructed DLI from fossil *Platanites*

**Figure 8:**
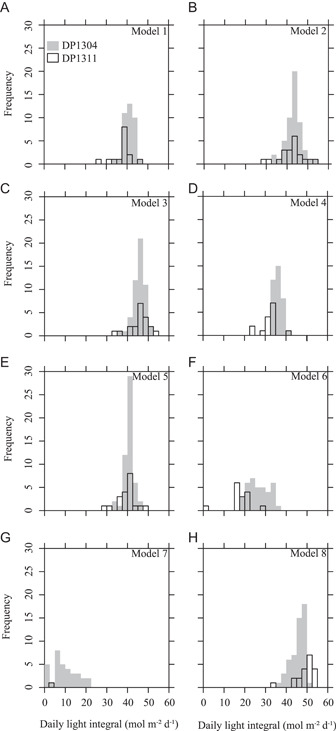
Histograms of reconstructed daily light integral (rDLI) of 72 individual *Platanites* fossil leaves from two sites (DP1304, DP1311) in the San Juan Basin, New Mexico based on eight regression models. Regression models include some combination of adaxial undulation index, adaxial cell area, and Δleaf. For full model details, see Table 2 in text. Note, we measured δ^13^C on a subset of fossil leaves (DP1304, *N* = 37; DP1311, *N* = 15); therefore, models that include this parameter (models 1, 4, 6, and 7) have lower frequencies of rDLI

Adaxial UI of *Platanites* was not significantly different between sites (*t* = –0.92, df = 24, *P* = 0.37) with a median value of 1.12 for both sites (Figure 3C). However, site DP1304 had significantly larger cell area than site DP1311 (*t* = 2.68, df = 27, *P* = 0.01; Figure 3A). δ^13^C_leaf_ was significantly lower (Figure 3A; *t* = 8.60, df = 43, *P* < 0.01) and Δleaf significantly higher (Figure 5C; *t* = –9.13, df = 43, *P* < 0.01) at site DP1311 compared to site DP1304. The values for Δleaf were consistent with those found in modern tropical rainforest (Diefendorf et al., 2010), which is similar to the reconstructed habitat based on reconstructed MAT and MAP estimates from the San Juan Basin (Flynn, 2020). Reconstructed DLI (rDLI) for both sites are dependent on the model used (Figure 8). Median rDLI estimates for most models (1, 2, 3, 5) range between ~39 to 46 mol m^−2^ d^−1^ with good agreement in rDLI between sites. Model 8 had the highest median rDLI (Figure 8H; DP1304 = 46.66 mol m^−2^ d^−1^, DP1311 = 50.33 mol m^−2^ d^−1^), while model 7 had the lowest median rDLI (Figure 8G; DP1304 = 7.94 mol m^−2^ d^−1^, DP1311 = –5.24 mol m^−2^ d^−1^).

## DISCUSSION

### Developing a proxy for paleo‐light environment from modern *Platanus*

We used two light experimental setups to quantify the effects of light intensity and quality on *Platanus occidentalis* and develop a predictive model for reconstructing daily light integral (DLI). We found that in modern *P. occidentalis* cell area, undulation index (UI), and δ^13^C_leaf_ all respond to DLI (Figures 3, 4, 6). Inclusion of all three traits in a linear regression model resulted in the best model for predicting DLI (Table 2). These results are similar to those of Cheesman et al. (2020), which found cell area, δ^13^C_leaf_, and a proxy for leaf mass per area to be the most consistent predictors of canopy position, and thus used in their predictive model of leaf area index (LAI). Notably, Cheesman et al. (2020) did not find UI to be a viable trait, perhaps because their predictive model was based on the averages for four species. Two of those species, *Myristica globosa* subsp. *muelleri* and *Cleistanthus myrianthus*, had no response of UI to LAI. However, if predictive models were made on a species level, UI would likely be included for the other two species tested: *Rockinghamia angustifolia* and *Endiandra microneural*. The work of Cheesman et al. (2020), therefore, demonstrates the importance of assessing species‐specific responses to light and the power of developing species‐specific models.

When developing a proxy to reconstruct past environments, it is important to consider uncertainties that can result from the inference spaces of the proxy, including the physical and biotic environment, genetics, and time (Jordan, 2011). Along these lines, we consider other factors, besides the light environment, that can influence the three traits measured in this study. A myriad of environmental (e.g., water availability, temperature) and plant attributes (e.g., phylogeny, growth form, age) can influence δ^13^C_leaf_ between 1‰ and 10‰ (see extensive reviews by Arens et al. [2000] and Diefendorf et al. [2010]). The use of LAI, rather than DLI (i.e., Dunn et al., 2015b; Cheesman et al., 2020), may partially account for some of these factors because LAI is known to vary with mean annual temperature, wetness index (a ratio of annual precipitation to potential evapotranspiration), and plant functional type (Iio et al., 2014). Nevertheless, even though carbon isotopes are correlated with light environment in modern systems (this study; Cheesman et al., 2020), applications to other environments or species may be problematic and require careful calibration. The method of Graham et al. (2014) avoids some of these pitfalls by using the range of δ^13^C_leaf_ values, under the assumption that absolute values may be subjected to other factors. The range in δ^13^C_leaf_ likely reflects the canopy effect at any one location, if enough leaves are sampled.

Like carbon isotopes, epidermal cell area has been shown to be affected by several factors in addition to light, including vapor pressure deficit (Carins Murphy et al., 2014), biome (Haworth and Raschi, 2014), ploidy (Beaulieu et al., 2008), and temperature (Arney, 1956; Pieters, 1974). However, the magnitude of the response (e.g., Carins Murphy et al., 2014) and the direction (e.g., Arney, 1956; Pieters, 1974) appear to be species‐specific. In contrast, there has been little research to analyze how other environmental factors influence UI. Of note, UI of *Betula nana* and *Betula pubescens* subsp. *czerepanovii* (N. I. Orlova) Hämet‐Ahti from Scandinavia is positively correlated to growing degree day (GDD), the cumulative seasonal sum of degrees above a threshold (Wagner‐Cremer et al., 2010; Ercan et al., 2020; Steinthorsdottir and Wagner‐Cremer, 2019; Ercan et al., 2021). Following the reasoning of Watson (1942), Wagner‐Cremer et al. (2010) hypothesized that increased GDD inhibits cuticle hardening, allowing for cells to grow larger and more sinuous (i.e., larger UI). If GDD influences UI in *P. occidentalis*, then the growth chamber experiments should have lower UI then expected, based on DLI alone, compared to the shade cloth experiment (7.5–9.1°C difference between experiments depending on treatment). However, we did not document this type of effect between the experimental setups (Figures 3, 4). While the environmental range sampled in this study is limited in scope, it is encouraging to find good agreement between the two experimental setups in this study for cell size and undulation (i.e., strong response in traits to DLI) even though there are disparate relative humidity and temperature environments (Table 1). Thus, our results suggest that GDD has limited influence on UI in *P. occidentalis*; however, more work is needed to evaluate the potential effect of GDD on UI.

The application of this proxy to fossil taxa must also contend with a phylogenetic framework, a critique outlined and described by Jordan (2011). The potential influence of phyologeny is especially true for our application to *Platanites*, an extinct genus within Platanaceae, which so far is known to comprise seven modern tree species (Nixon and Poole, 2003). Traditional stomatal proxy methods (i.e., stomatal index and stomatal ratio) provide some guidance on how to account for potential phylogenetic effects because those methods often involve a single species or morphotype. So far, this genetic component has been explored by either (1) comparing the response of closely related, multiple species (e.g., Barclay et al., 2010; Haworth et al., 2010; Steinthorsdottir et al., 2019) and/or (2) using the nearest living relative or closest ecological equivalent (e.g., McElwain, 1998; Royer, 2003; Barclay et al., 2010; Steinthorsdottir et al., 2019). As it extends to Platanaceae, Royer (2003) reconstructed CO_2_ from the Paleocene using the stomatal ratio method by comparing stomatal indices of *P. guillelmae* to those of modern *Platanus occidentalis*, *P. orientalis*, and their hybrid *P*.×*acerfolia*. Extant species were selected based on similarities in morphology and ecology to both *Platanites raynoldsii* Newberry and *P. guillelmae* (Royer et al., 2003).

We argue that based on the response to other environmental variables and incongruence of fossil data to the modern calibration, cell area and δ^13^C_leaf_ cannot be used as a quantitative approach to reconstruct DLI based on a single taxon, *P. occidentalis*. Instead, the range of the data may possibly be used as a qualitative approach. However, based on our data, UI for *P. occidentalis* appears to be a reliable proxy to quantitatively constrain DLI from the fossil record (model 2). In particular, we have demonstrated that (1) UI in *P. occidentalis* can be quantified over a range of light values, (2) light quantity not light quality drives UI, (3) different temperature and humidity regimes had little effect on UI, and (4) the abaxial and adaxial surface had the same UI light response, meaning either surface can be used in DLI reconstruction. Additionally, the fossil UI estimates fall within the calibration space of the model (unlike that for cell area). However, we note that further experimental work that looks at UI and isolated environmental variables (e.g., temperature/GDD, humidity, and VPD) as well as evaluating the UI response within a single genus or family, could help clarify how environmental variables and/or phylogenetics control the response.

### High rDLI values indicate predominately sun leaves

A comparison of Δleaf, UI, and cell area between the data from our fossil and modern samples appear to provide conflicting results. For example, the fossil Δleaf range (DP1304, 20.1–23.6‰) most closely matches the Δleaf range found in the 90% shade cloth, while site DP1311 values are, on average, higher than all values from the shade cloth experiment (Figure 6C). Additionally, average adaxial cell area is ~43–53% lower than the average area from the no shade cloth treatment depending on fossil locality (Figure 3A). The reduction in adaxial cell area seen here is more than what would be expected from cuticle shrinkage alone (Cleal and Shute, 2007). Therefore, Δleaf would indicate a closed canopy environment, as seen by the implausibly low and negative rDLI values of model 7 (Figure 8G), while cell area and UI would indicate an open environment as indicated by high rDLI values (model 2, 5, 8).

The fossilization process includes several steps that can potentially bias the fossil record by preferentially preserving sun leaves (though see Guignard et al., 2001; Bush et al., 2017). Upper canopy leaves contribute the most litter production (Osada et al., 2001) and are more likely to survive transport to deposition (Spicer, 1981). Additionally, these leaves are known to have higher leaf mass per area (Koch et al., 2004; Sack et al., 2006) and thicker cuticles (Osborn and Taylor, 1990) compared to shade leaves, which can increase their preservation potential. Based on these taphonomic processes, we find it highly unlikely that Δleaf values record only leaves from deep within the canopy. More likely, other variables are influencing δ^13^C_leaf_, causing an offset between the calibration data set and the fossils. In addition, when δ^13^C_leaf_ is compared to adaxial UI, there is no correlation between UI and δ^13^C_leaf_ (Figure 7A), consistent with the relationship between adaxial UI and δ^13^C_leaf_ at the light treatment level from the modern experiments (Figure 7B). The relationship between δ^13^C and adaxial UI of our fossils suggests that our fossil assemblages are from one light environment, rather than a range of light environments. However, we do note that the sample sizes, especially for site DP1311 (*N* = 15) are not adequate to capture the full range of light environments as outlined in Graham et al. (2014) where 50 or more leaves sampled randomly are recommended.

On the basis of UI (model 2), our rDLI values from *Platanites*, with median values of 43.63–43.77 mol m^−2^ d^−1^, are within the range of DLI values found within the United States (Faust and Logan, 2018) and globally (Poorter et al. 2019), which range from ~0 to 65 mol m^−2^ d^−1^ depending on season, latitude, and cloudiness (Faust and Logan, 2018; Poorter et al., 2019). Additionally, the range of rDLI at each site is narrow, between 32.94 and 52.65 mol m^−2^ d^−1^ for site DP1304 and 28.45–54.01 mol m^−2^ d^−1^ for site DP1311. These results suggest that we are not sampling leaves from deep within the canopy, as light transmission may be reduced by up to 50% in the first few meters of the canopy and by as much as 95–99% by the forest floor (Ellsworth and Reich, 1993; Yang et al., 1993). Therefore, the use of these leaves for reconstructing paleoclimate (i.e., temperature and atmospheric CO_2_) likely avoids biases that could result from large changes in light environments. In addition, high DLI values are associated with an increase in in photosynthesis and transpiration (Poorter et al., 2019), which has important implications for carbon and water vapor cycling through Earth history (e.g., White et al., 2020).

### Canopy architecture of early Paleocene forests

**Figure 9:**
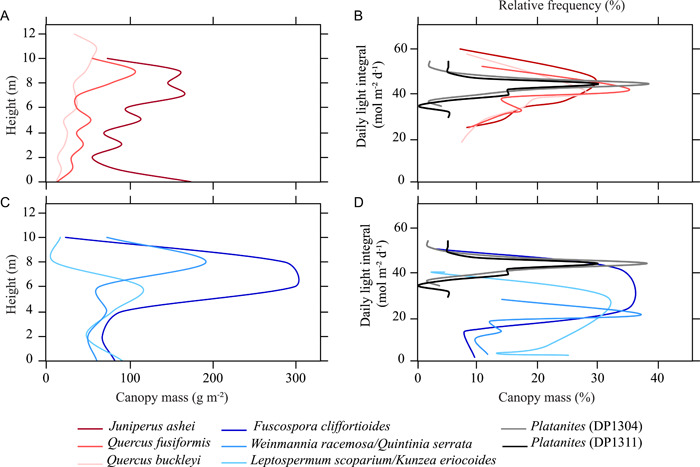
(A, C) Mean canopy mass values by height for Texas (Thomas et al., 2016) and New Zealand (White and Scott, 2006) flora. (B, D) Comparison of the relative frequency (%) of reconstructed daily light integral (DLI) from *Platanites* to percent canopy mass of known DLI. Methods for determining DLI for Texas and New Zealand flora can be found in Appendix S4

Because canopy architecture determines light interception through a forest canopy (Kira et al., 1969; Sampson and Smith, 1993), the rDLI values likely represent the position of an individual leaf within the forest stand. Canopy architecture is reflected in the vertical distribution of leaves, represented as canopy leaf mass. Whereas single cohort stands may have foliage that is aggregated toward the top of the canopy, multiple‐cohort stands have foliage distributed broadly across the entirety of the vegetation height. For example, the percentage of canopy mass of previously studied Texas (Thomas et al., 2016) and New Zealand (White and Scott, 2006) floras show most of the Texas woodland types have the highest proportion of leaves at the top of the canopy, particularly for *Quercus fusiformis* and *Q. buckleyi*. For the New Zealand forests, there is a broader dispersion of canopy mass across height (Figure 9C). With these canopy profiles, the estimated DLI of Texas woodlands show that the most light is intercepted at the top of the canopy where light penetrates the New Zealand forests more deeply (Figure 9). The *Platanites* rDLI distributions (model 2) show similarity to the distribution of DLI found in stands of *Q. fusiformis* and *Q. buckleyi*, which are reported to be single‐cohort, even‐aged stands (Thomas et al., 2016) (Figure 9B).

**Figure 10:**
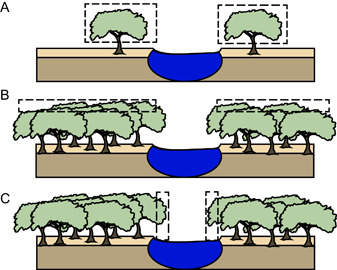
Schematic representation of alternative interpretations of canopy architecture for *Platanites*. Boxes indicate the likely source of fossil leaves. (A) Sparse canopy. (B) Dense canopy but fossilized leaves are preferentially preserved from the top of the canopy. (C) Dense canopy but fossilized leaves are from preferentially preserved from the forest edges

There are three main alternative possibilities in interpreting canopy architecture in the context of our high rDLI values (Figure 10). First, there was a sparse canopy, with isolated *Platanites* trees (Figure 10A). The entire canopy of these trees therefore receives sunlight, and this crown is preferentially preserved within the fossil record. Based on the diversity of morphotypes and the abundance of fossils leaves recorded at these locations (Flynn, 2020), this scenario seems unlikely. Second, there was a dense forest, but only part of the upper canopy is preserved (Figure 10B). In this scenario, the narrow range of rDLI reflects taphonomic process preferentially preserving sun leaves from the first few meters of the canopy. Third, there was a dense canopy, but fossilized leaves were from the forest edge, perhaps along the edge of a river or pond (Figure 10C). Modern (Everson and Boucher, 1998) and fossil Platanaceae (Royer et al., 2003) are commonly found in riparian environments. Indeed, the sedimentological evidence that our fossils are from crevasse splays lends support to the idea of a disturbed environment, with *Platanites* trees living along the edge of streams and rivers where there are openings in the canopy. If scenario two or three were true, then the distribution of rDLI would be biased by sunlit leaves. This in turn, would call into question the interpretation of canopy mass within the upper crown of the forest stand. Resolving which of these alternative interpretations is most likely has important implications for understanding paleo‐canopy architecture, which in turn, is critical for understanding different aspects of Earth's climate (Bastable et al., 1993; Kala et al., 2014), ecological interactions (Kay et al., 1997; Asner et al., 2003; Dunn et al., 2015b), and animal evolution (Field et al., 2018).

## CONCLUSIONS

This study examined how several traits (UI, cell area, δ^13^C_leaf_) of *P. occidentalis* varied based on two different experimental setups. We find that all traits showed a significant response to DLI. However, UI alone was the best trait to use for reconstructing DLI for *Platanites* in the geological record. Due to multiple biotic and abiotic factors known to influence δ^13^C, along with the mismatch between modern and fossil cell area, these traits should be used only as a qualitative supplement to UI. Importantly, our work on UI shows that it is (1) a quantitative measurement over a continuous light interval, (2) influenced by light quantity not light quality, and (3) robust to different temperature and humidity values. Based on UI, we found high (~43 mol m^−2^ d^−1^) median rDLI values from two early Paleocene sites in the San Juan Basin, New Mexico. The distribution of high DLI values from fossil leaves may provide information on canopy architecture; indicating that either most of the canopy mass is within the upper portion of the crown or that leaves exposed to more sunlight are being preferentially preserved. In addition to further experimental work that looks at UI with respect to other environmental variables, as well as phylogeny, more work is needed to address the role of depositional environments in reconstructing DLI, such as sampling locations that are known to have a closed canopy.

## AUTHOR CONTRIBUTIONS

J.N.M, D.J.P., R.E.D, R.S.B., and J.D.W. conceived and developed the project. J.N.M., A.G.F., L.L.R.K, J.D.W., B.W.B, and B.Z. collected and processed the data. J.N.M and D.J.P wrote the manuscript with input from all authors.

## Supporting information


**Appendix S1**. Shade cloth experiment study location.

**Appendix S2**. Comparison of average atmospheric δ^13^C from Mauna Loa, Hawaii and Moody, Texas.

**Appendix S3**. Statistics for Student's *t*‐test, ANOVA, regression models, and model results for reconstructed daily light integral.

**Appendix S4**. Daily light integral for Texas and New Zealand stands.

## Data Availability

The data set analyzed during the current study is available in the Texas Data Repository (https://doi.org/10.18738/T8/DGHGJA).
