# Supplementary figures and images for: Quantifying the effect of shade on cuticle morphology and carbon isotopes of sycamores: present and past
Source: Am J Bot. 2021 Dec 31;108(12):2435–51. doi: 10.1002/ajb2.1772 (PMC9306692; doi:10.1002/ajb2.1772)

Appendix S1. Shade cloth experiment from the Waco Wetlands, Waco, Tx. Scale bar is 3.0 m.

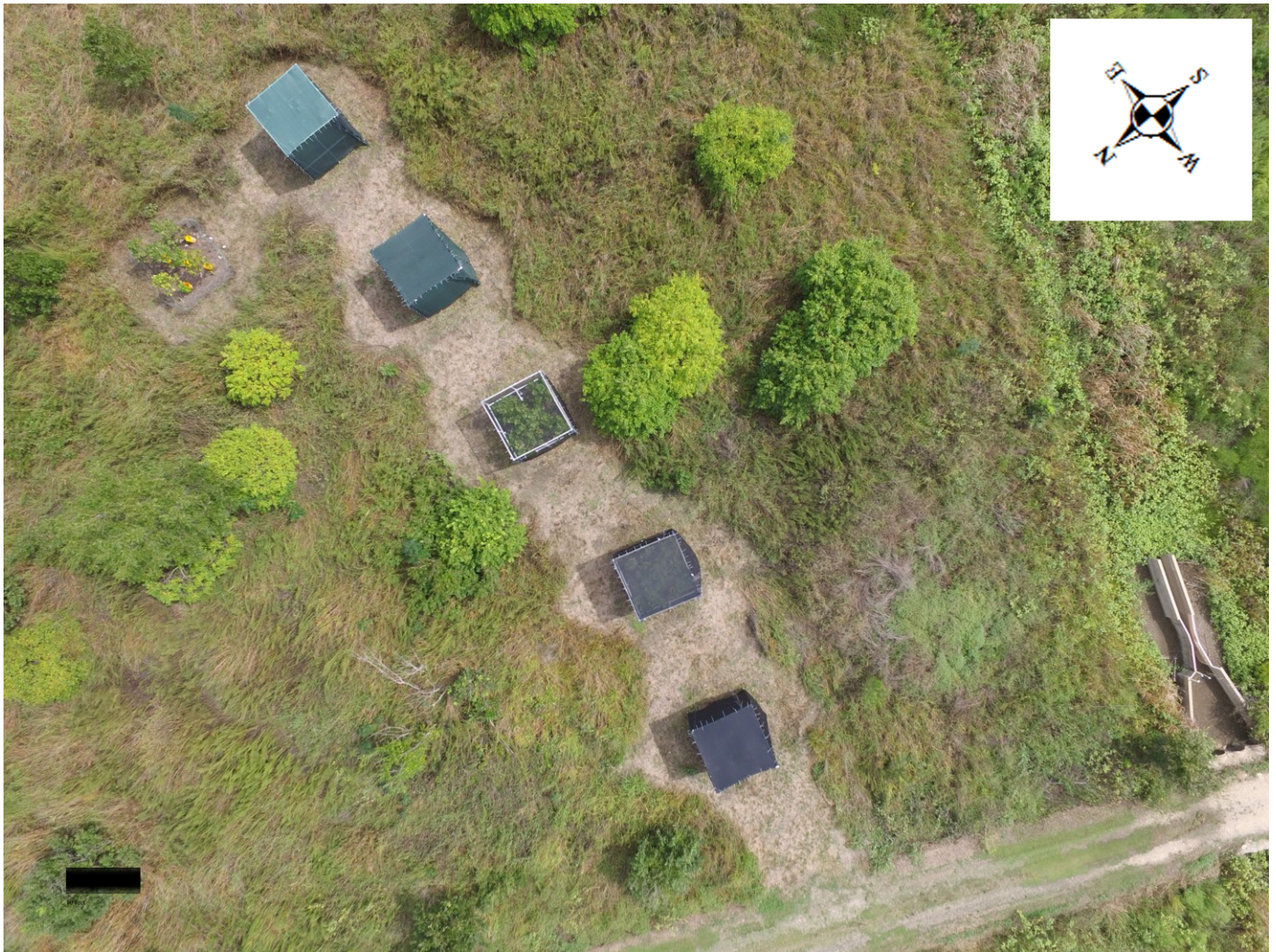

Supplement: Supplementary file 1 — Appendix S1. Shade cloth experiment study location. [file AJB2-108-2435-s004.pdf]
