# Supplementary material for: Quantifying the effect of shade on cuticle morphology and carbon isotopes of sycamores: present and past
Source: Am J Bot. 2021 Dec 31;108(12):2435–51. doi: 10.1002/ajb2.1772 (PMC9306692; doi:10.1002/ajb2.1772)
Supplement: Supplementary file 2 — Appendix S2. Comparison of average atmospheric δ13C from Mauna Loa, Hawaii and Moody, Texas. [file AJB2-108-2435-s003.docx]

Milligan et al. – American Journal of Botany 2021 – Appendix S2

Appendix S2. Average atmospheric δ^13^C from Mauna Loa, Hawaii (MLO) and Moody, Texas (WKT) for the months March-August. The Moody site is located ~44 km away from the shade cloth experiment. Data from 2001-2010 are from White et al. (2015) while data from 2018 are from Keeling et al. (2005).

| Year | MLO  (‰) | WKT  (‰) | Mean error rate  (%) |
| --- | --- | --- | --- |
| 2001 | -8.13 | -8.41 | 3.32 |
| 2002 | -8.15 | -8.24 | 1.02 |
| 2003 | -8.23 | -8.31 | 0.88 |
| 2004 | -8.27 | -8.35 | 0.99 |
| 2005 | -8.30 | -8.35 | 0.66 |
| 2006 | -8.32 | -8.35 | 0.32 |
| 2007 | -8.32 | -8.35 | 0.37 |
| 2008 | -8.32 | -8.35 | 0.39 |
| 2009 | -8.35 | -8.38 | 0.47 |
| 2010 | -8.39 | -8.42 | 0.31 |
| 2018 | -8.59 | - | - |

White, J.W.C., B.H. Vaughn, and S.E. Michel (2015), University of Colorado, Institute of Arctic and Alpine Research (INSTAAR), Stable Isotopic Composition of Atmospheric Carbon Dioxide (13C and 18O) from the NOAA ESRL Carbon Cycle Cooperative Global Air Sampling Network, 1990-2014, Version: 2015-10-26, Path: ftp://aftp.cmdl.noaa.gov/data/trace_gases/co2c13/flask/.

D. Keeling, S. C. Piper, R. B. Bacastow, M. Wahlen, T. P. Whorf, M. Heimann, and H. A. Meijer, Exchanges of atmospheric CO_2_ and ^13^CO_2_ with the terrestrial biosphere and oceans from 1978 to 2000. I. Global aspects, SIO Reference Series, No. 01-06, Scripps Institution of Oceanography, San Diego, 88 pages, 2001.
