# Supplementary material for: Quantifying the effect of shade on cuticle morphology and carbon isotopes of sycamores: present and past
Source: Am J Bot. 2021 Dec 31;108(12):2435–51. doi: 10.1002/ajb2.1772 (PMC9306692; doi:10.1002/ajb2.1772)
Supplement: Supplementary file 4 — Appendix S4. Daily light integral for Texas and New Zealand stands. [file AJB2-108-2435-s002.docx]

Milligan et al. – American Journal of Botany 2021 – Appendix S4

For the Texas woodlands, annual, average, daily irradiance was estimated by calculating the daily instantaneous irradiance (J m^-2^ s^-1^) daylength (seconds) from the MT-CLIM model (Running et al., 1987) utilizing minimum and maximum temperature and daily precipitation as model inputs. For each day, daily irradiance was calculated by multiplying the instantaneous irradiance by the daylength that was then averaged for the year and converted in a photon flux density value (μmol m^-2^ s^-1^).

For New Zealand, monthly, daily average short-wave radiation (MJ m^-2^ day^-1^), were derived from spatially interpolated meteorological data for measured locations (J.R. Leathwick and R.T.T. Stephens, unpublished data) that averaged for all months and converted into photo flux density.

From both forests, DLI was calculated by multiplying the annual irradiance by average transmissivity measured at 2 m vertical distances in all stands (See White and Scott (2006) for details).

Running, S. W., R. R. Nemani, and R. D. Hungerford.1987. Extrapolation of synoptic meteorological data in mountainous terrain and its use for simulating forest evapotranspiration and photosynthesis. *Canadian Journal of Forest Research* 17: 472-483.

White, J. D., and N. A. Scott. 2006. Specific leaf area and nitrogen distribution in New Zealand forests: Species independently respond to intercepted light. *Forest Ecology and Management* 226: 319-329.
